# Polarization Properties and Polarization Depth Profiles of (Cd:Zn)S/P(VDF-TrFE) Composite Films in Dependence of Optical Excitation

**DOI:** 10.3390/polym10111205

**Published:** 2018-10-30

**Authors:** Sebastian Engel, David Smykalla, Bernd Ploss, Stephan Gräf, Frank A. Müller

**Affiliations:** 1 Otto Schott Institute of Materials Research (OSIM), Friedrich Schiller University Jena, Löbdergraben 32, 07743 Jena, Germany; stephan.graef@uni-jena.de (S.G.); frank.mueller@uni-jena.de (F.A.M.); 2 Department of SciTec, University of Applied Sciences Jena, Carl-Zeiss-Promenade 2, 07745 Jena, Germany; david.smykalla@eah-jena.de (D.S.); bernd.ploss@eah-jena.de (B.P.); 3 Jena Center for Soft Matter (JCSM), Friedrich Schiller University Jena, Philosophenweg 7, 07743 Jena, Germany; 4 Center for Energy and Environmental Chemistry (CEEC), Friedrich Schiller University Jena, Philosophenweg 7a, 07743 Jena, Germany

**Keywords:** composite, ferroelectric polymer, semiconductor, optical excitation

## Abstract

The influence of optical excitation intensity on the electrical, ferroelectric and pyroelectric properties of ferroelectric-semiconductor-composites was investigated. For this purpose, composite thin films consisting of poly(vinylidene fluoride-*co*-trifluoroethylene) and 10 vol % (Cd:Zn)S particles with a thickness of 34 µm were fabricated. The samples were used to measure the absolute pyrocoefficient and to determine the relative pyroelectric depth profile using Laser Intensity Modulated Method. It was shown that a polarization of the samples without an optical excitation at the utilized relatively small peak-to-peak voltages could not be verified by the Sawyer–Tower circuit and the measurement setup of the pyroelectric coefficient, respectively. Both remanent polarization and pyroelectric coefficients increased with increasing optical excitation intensity during poling as well as increasing peak-to-peak voltage. The pyrocoefficient shows a temporal decay in the first hours after poling. The specific heat and thermal conductivity or the thermal diffusivity are required for the calibration of the pyroelectric depth profile. Rule of mixture and photo-acoustic investigations proved that the thermal properties of the utilized composites do not differ significantly from those of the pristine polymer. Based on the pyroelectric depth profile which is proportional to the polarization profile, the existing “three phase model” has been extended to generate a replacement circuit diagram, explaining the local polarization due to the optical excitation dependency for both local resistivity and local field strength.

## 1. Introduction

Ferroelectrics made of polymers are characterized by advantageous compared to their ceramic competitors due to their low processing temperatures, large electrical resistivity, and their high mechanical flexibility. They are of particular interest for engineering flexible electronic devices such as energy harvesting systems, memory devices and sensors [1,2,3,4,5,6,7,8,9]. A specific example of this type of material is poly(vinylidene fluoride-*co*-trifluoroethylene) P(VDF-TrFE), which exhibits a well-ordered, polar, and ferroelectric *β*-phase structure [10,11]. For a TrFE content in the range of 20–45 mol %, P(VDF-TrFE) crystallizes directly in this ferroelectric *β*-phase, independent of processing routes or post-treatment procedures [12]. In addition, the solubility of P(VDF-TrFE) in various solvents makes the material attractive for flexible electronics fabricated by spin coating [13], dip coating [14], and screen printing [15]. Beyond, the suitability to disperse (nano-)particles into the polymer allows to tailor the electrical and ferroelectric materials properties. In this way, polymer-ceramic nanocomposites could be synthesized which are able to detect either pressure or temperature [16,17]. Furthermore, it has been found that the addition of ceramic or metallic nanoparticles can enhance the ferroelectric properties of P(VDF-TrFE) [18,19,20,21,22,23,24]. Separate poling of the inclusions and of the matrix of a ferroelectric composite with 0–3 connectivity usually requires a change of the matrix conductivity [17]. An increase in matrix conductivity can be achieved by increasing the temperature [16]. However, a more specific adjustment of the matrix conductivity is of great interest for the polarization of inclusions and also for specific detector properties [25,26,27]. Recently, the tailored conductivity adjustment via photoexcitation was realized by composites of P(VDF-TrFE) with semiconductor particles [28]. It was determined that the photoinduced conductivity influences the pyro- and thus also the piezoelectric performance of the composites and their polarization behaviors. In addition, Fourier transformed infrared spectroscopy and X-ray diffraction (XRD) analyses showed that the dispersed (Cd:Zn)S particles have a negligible influence on the internal crystalline (ß-phase) and amorphous (semi-crystalline) structure of the P(VDF-TrFE) matrix [28]. In order to gain a deeper understanding of the proposed “three phase model” [28], our present study focus on polarization investigations and pyroelectric measurements of P(VDF-TrFE)-(Cd:Zn)S composites in dependence of an on optical excitation. In particular, the Laser Intensity Modulation Method (LIMM) [29,30] allows the characterization of the bulk of the composite by measuring the pyroelectric depth profile and thus also the spatial polarization profile in dependence of an optical excitation during poling. The reliable application of this method for the semi-transparent electrodes was demonstrated by optical characterizations in [28], caused by the very low transmitted light power through the sample which results in no additional heat source at the back electrode. In detail the negligible distortion is smaller than 0.5% due to the combination of the very small transparency of the front electrode and the high scattering without absorption at the utilized LIMM-laser wavelength of *λ* = 685 nm of the composite itself.

## 2. Materials and Methods

### 2.1. Sample Preparation

Based on the results of our previous study [28], a composite with a (Cd:Zn)S particle (<500 nm, Kremer Pigmente, Aichstetten, Germany) concentration of 10 vol % in 70/30 Poly(vinylidene fluoride-*co*-trifluoroethylene) [P(VDF-TrFE)] (molecular weight of 450,000 g/mol, Piezotech Arkema, Pierre-Benite Cedex, France) was fabricated. This volume fraction provides the maximum impact of optical excitation on the ferro- and pyroelectric properties of the composite sample. The composite samples were prepared by dispersing 100 mg 70/30 P(VDF-TrFE) and 27 mg (Cd:Zn)S powder in a 100 mL methyl ethyl ketone (Carl Roth, Karlsruhe, Germany) ultrasonic bath followed by magnetic stirring at 50 °C for 180 min. Composite foils with a final thickness of 34 µm were fabricated by evaporation of the methyl ethyl ketone from the prepared solution in a petri dish and subsequent compression molding of 4 superimposed composite layers for 3 min at 170 °C and 30 kN. This ensures homogenous composite samples concerning thickness, surface quality, and (Cd:Zn)S particle distribution. Additional annealing steps were not performed. XRD-characterization of both samples led to the same spectra as those of our previous work [28] at the corresponding particle concentration. According to Ref. [31], the diffraction curves were separated into a crystalline and an amorphous peak, and the ratio of the area of the crystalline diffraction intensity over the total coherent scattering was considered as the degree of crystallinity *X*_C_. The value of *X*_C_ for the P(VDFTrFE)-matrix of both composite samples was calculated to be about 75%. Subsequently, the semi-transparent circular gold electrodes were deposited on the top surface (thickness: 30 nm, diameter: 6 mm) and on the bottom surface of the composite foils (thickness: 30 nm, diameter: 8 mm) by sputtering to enable electrical contacting and to allow the same optical excitation from the top and the bottom surface. Finally, one of the composite foils, subsequently referred as sample 1, was fixed on a transfer aluminum foil to minimize the mechanical stress between each polarization and pyroelectric measurement step resulting from the required movements between these two experimental setups. Another composite foil, subsequently referred as sample 2, was bonded to a special circuit board with well-defined electrical contacts, which was adjusted to the Laser Intensity Modulated Method (LIMM)-setup in order to characterize the pyroelectric depth profile.

### 2.2. Electrical, Ferroelectric and Pyroelectric Properties

The electrical and ferroelectric properties of the composite samples were investigated by performing I-V and polarization measurements using the experimental setups described in [28]. This setups were supplemented by an optical excitation source in terms of a LED (LXML-PB01-0023, LUMILEDS, Amsterdam, Netherlands) with a central wavelength of 470 nm and bandwidth FWHM of 20 nm. Using a power control unit (Laboratory Power Supply PS 2403d, Voltcraft, Wernberg-Köblitz, Germany), the intensity of the LED was adjusted between 0 and 12 mW/mm^2^ for sample 1. For sample 2, the maximum light intensity was limited to 8 mW/mm^2^ because of the special circuit board with its well-defined electrical contacts resulting in larger distance between the LED and the sample foil. Before each measurement, the optical power at the respective central wavelength was set using a power meter (S130VC, Thorlabs, Dachau/München, Germany). An optical density filter (NE10A, Thorlabs, Dachau/München, Germany) was utilized to prevent an overload and was taken into account for the determination of the real power.

The ferroelectric hysteresis loops of the composite foils were recorded utilizing a Sawyer–Tower circuit at a frequency of 10 Hz and peak-to-peak voltages between 2.4 and 3.8 kV. The capacity of the reference capacitor was 1 µF. One polarization step consisted of 50 loops with a triangular high voltage. To investigate the influence of an optical excitation on the ferroelectric properties of the composite samples during the polarization loops, the intensity of the 470 nm LED was tuned from 0 to 12 mW/mm^2^ (sample 1) and from 0 to 8 mW/mm^2^ (sample 2), respectively.

**Figure 1:**
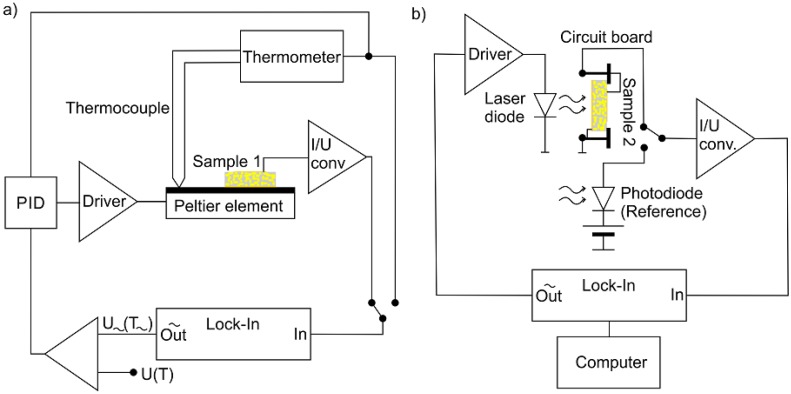
Schematic illustration of the experimental setup (**a**) for the dynamic measurement of the pyroelectric coefficient and (**b**) for the LIMM-measurement.

An ac method [32,33] was used to measure the pyroelectric coefficient of sample 1 (Figure 1a). At a certain mean temperature $T_{0}=28\;{}^{\circ}C$, the sample temperature was sinusoidally modulated [$T_{m}\left(t\right)=T_{0}+T_{\sim }\cdot \sin\left(2\pi ft\right)$] with a frequency *f* = 23.5 mHz and an amplitude $T_{\sim }=1\;K$ using a Peltier element (TEC1-12706, Roßmann Electronic GmbH, Dießen am Ammersee, Germany) controlled by a proportional integral differential (PID) regulator [17]. The pyroelectric current signal was amplified with a current-to-voltage converter (I/U converter). The 90° out-of-phase component of the current with respect to $T_{m}\left(t\right)$ (i.e., in phase with temporal derivative $dT_{m}\left(t\right)/dt$)) was measured with a lock-in amplifier. In order to ensure a homogenous temperature modulation of the composite sample during the pyroelectric measurement after each polarization step, the surface of the Peltier element and the whole composite sample as well as the transfer aluminum foil were covered with a thin silicon oil film (Silikon Öl B5, Silikon Profis, QUAX GmbH, Otzberg, Germany). Its optical transparency between 200 and 1000 nm allows the optical excitation during each polarization step between two pyroelectric measurement steps. In addition the silicon oil film prevents an increase of the sample temperature caused by the optical excitation.

### 2.3. Characterization of the Pyroelectric Depth Profile

For pyroelectric materials, the depth profile of the pyroelectric coefficient and therefore the spatial polarization profile can be determined by the Laser Intensity Modulated Method (LIMM) illustrated in Figure 1b [29,30,34,35]. The sinusoidally modulated intensity of a laser diode (*λ* = 685 nm) was used to irradiate the electrode of sample 2 in the frequency range from 10 to 10.4 × 10^5^ Hz. This was performed for both sides of the foil to generate the pyroelectric depth profile across the whole sample thickness [30]. The modulated power absorbed in an electrode generates a thermal wave, which penetrates into the composite-film. The attenuation and therefore the penetration depth of these thermal waves decreases with increasing frequency. Resulting from the pyroelectric effect, the local temperature causes a pyroelectric response, which is a convolution of the pyroelectric coefficient distribution and the temperature profile across the sample. Consequently, the frequency and polarization profile dependent pyroelectric current between ground and top electrode was measured phase sensitive by a lock-in amplifier. The distribution of the pyroelectric coefficient p(z) and thus the polarization profile across the thickness P(z) of the sample can be approximated from the measured frequency dependency of the pyroelectric current *I*(*ω*) [30], which is proportional to the reciprocal of the specific heat per volume of the sample material:

(1) $$I_{\sim }\;\approx \;\frac{1}{c_{S}\cdot \rho_{S}}$$

Here, $c_{s}$ refers to the specific heat capacity and $\rho_{s}$ is the mass density of the sample. Both values can be calculated for the composites from those of the components and their volume ratios by the rule of mixtures. With c=0.468 J/g·K and c=0.380 J/g·K for ZnS and CdS [36] and their densities of $\rho =4.089\;g/{\mathrm{cm}}^{3}$ [37] and $\rho =4.82\;g/{\mathrm{cm}}^{3}$ [38] follows $c\cdot \rho =1.913\;J/{\mathrm{cm}}^{3}\cdot K$ and $c\cdot \rho =1.83\;J/{\mathrm{cm}}^{3}\cdot K$, respectively. Consequently, c·ρ of composites with up to 15 vol % (Cd:Zn)S differs from that of P(VDF-TrFE) with $1.99\;J/{\mathrm{cm}}^{3}\cdot K$ [39] by not more than 2%. Therefore, absolute values of the pyroelectric profiles of the various composites and the pristine copolymer which are recorded under equivalent experimental conditions in arbitrary units, can be compared directly. One subject of the current work was to investigate the influence of optical excitation of (Cd:Zn)S particles on the polarization behavior and thus the spatial polarization profile of the composite film. Since the composite sample itself and the absorption behavior of the electrodes and their positioning in the experimental setup remain the same between the polarization steps, the calculated polarization profiles are comparable to each other. Additionally, considering the measured remanent polarization of sample 1 and 2 and the global pyroelectric coefficient of sample 1, the measurement of the surface temperatures for an absolute calibration of the pyroelectric profiles is not required. The assignment of the pyroelectric current measured at the angular frequency *ω* to a point at a distance *x_r_* from the surface is determined by the thermal diffusivity *D* [30]:

(2) $$x_{r}=\sqrt{2D/\omega }$$

Thus the photo-acoustic characterization was applied to compare the thermal transport in the composites and the pristine copolymer.

### 2.4. Photo-Acoustic Characterization of the Thermal Properties

**Figure 2:**
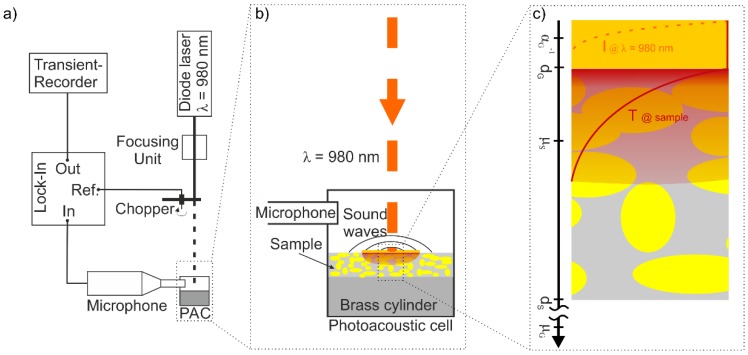
Schematic illustration of (**a**) the experimental setup used for photoacoustic measurements including (**b**) the photoacoustic cell and (**c**) the special case of an optically opaque and thermally thin solid Rosencwaig and Gersho (RG)-theory [44]).

Photo-acoustic (PA) methods like the photo-acoustic spectroscopy allow to detect that part of the absorbed light energy, which is converted into heat after optical excitation. For solids, the heat can be measured indirectly using acoustic sensors via the volume expansion of the bulk medium (piezoelectric detection technique [40]) or of the surrounding atmosphere (gas-microphone). For the latter, the sample is placed inside a gas tight chamber with an optical transparent window. To minimize a distortion of the PA-signal caused by a background PA-signal from the PA-cell itself, fused silica (Infrasil 301, Heraeus, Hanau, Germany) was used not only as optical window material but also as a transparent construction material [41,42]. Thus, scattered modulated excitation radiation passes the chamber wall without creating a separate PA-signal [43]. According to the theory of Rosencwaig and Gersho (RG-Theory) [44], the sample properties can be determined from the intensity of a PA signal induced by the excitation with light modulated at a certain frequency. Here, the optical absorption length $\alpha^{-1}$, the thermal diffusion length μ and the thickness of the sample d are of particular importance. In order to reveal the effect of the semiconductor particles on the thermal properties of the composite, samples with particle concentrations of 0, 0.1, 1, 5, 10 and 15 vol % were analyzed based on our previous study [28]. Thus, in contrast to the LIMM measurement various samples with different optical properties were utilized [28]. To avoid the influence based on these different optical properties by e.g., different transmission or scattering, gold layers with a thickness of ~200 nm were deposited by sputtering. This also excludes the influence of different transmitted parts of the light energy between the individual samples due to small fluctuations of the gold layer thickness. Beyond, the photo-acoustic measurements of the composite samples are not dependent on their optical properties. This is due to the fact that the gold layers act as optically opaque samples, which were heated up by the absorbed part of the modulated light intensity with a certain frequency. Since the gold layer is the same for all samples, the PA-signal amplitude (SA) between the composite samples is only influenced by their thermal properties. According to the RG-theory, the thin sputtered gold layer of $d_{G}=200\;\mathrm{nm}$ with an optical penetration depth of $\alpha_{G}^{-1}<200\;\mathrm{nm}$ [45] and high thermal diffusion lengths $\mu_{G}=\sqrt{2\cdot \kappa_{G}/\omega \cdot \rho_{G}\cdot c_{G}}$ ($\mu_{G}=160\;\mu m$ for 1500 Hz and $\mu_{G}=90\;\mu m$ for 4500 Hz with $c_{G}=132\;J/\left(\mathrm{kg}\cdot K\right)$ [46], $\rho_{G}=19.3\;g/{\mathrm{cm}}^{3}$ [46] and $\kappa_{G}=317\;W/(m\cdot K)$ [47]) fulfills the special case of an optically opaque and thermally thin solid ($\alpha_{G}^{-1}<d_{G}$, $d_{G}<\mu_{G}$, Figure 2b) on a thermally thick composite substrate [44]. In this special case, the *SA* depends on the thermal properties of the substrate, which are represented by the composite samples with different volume fractions of semiconductor particles [44]:

(3) $$SA≅\frac{\mu_{Gas}}{2}\cdot \left(\frac{\mu_{S}}{\kappa_{S}}\right)\cdot G$$

Here, $\mu_{Gas}$ refers to the thermal diffusion length of the surrounding gas and *G* is a constant of the utilized PA-setup. On the basis of $\mu_{Gas}=\sqrt{2\cdot \kappa_{Gas}/\omega \cdot \rho_{Gas}\cdot c_{Gas}}$, which depends only on ω, the factor $\mu_{Gas}\cdot G$ remains the same under the same measurement conditions, regardless of the sample at a certain frequency. In this way, at a certain frequency *SA* is determined only by the thermal properties of the substrate where $\kappa_{S}$ is the thermal conductivity of the sample:

(4) $$SA\sim \sqrt{\frac{1}{c_{s}\cdot \rho_{s}\cdot \kappa_{s}}}\;\;\;\;\;=\frac{1}{c_{s}\cdot \rho_{s}}\;\;\sqrt{\frac{1}{D_{S}}}$$

Consequently in this special case, the PA-measurements can be used to determine whether the particle concentration has an influence on the thermal diffusivity $D_{S}$ of the samples in comparison to the pristine P(VDF-TrFE) 70/30, for which $D_{S}=1.09\cdot 10^{-3}{\;\mathrm{cm}}^{2}/s$ [39]. The specific PA measurements were carried out using an experimental setup described in [48] (Figure 2a). This setup was supplemented by a polished brass cylinder (4 mm thickness, 8 mm diameter, Figure 2b), placed inside the photoacoustic cell to decrease the volume of the gas tight chamber resulting in an increased SA. The chopper (MC 2000, Thorlabs GmbH, Dachau/München, Germany) used for light modulation was set to frequencies *f* = 1500 Hz and *f* = 4500 Hz, respectively. Considering the thermal properties of pristine P(VDF-TrFE) 70/30, the first one allows the detection of the thermal properties of the composites up to a higher thermal diffusion length $\mu_{S}=\sqrt{2\cdot D_{S}/\omega }$ around 5 µm without influencing the thermal properties due to the polished brass cylinder. In order to ensure no influence due to thermal properties of the polished brass cylinder and to confirm the results of the first PA measurements, the second PA measurements with *f* = 4500 Hz and therefore a thermal diffusion length $\mu_{S}$ of around 2.7 µm were realized.

## 3. Results

### 3.1. Electrical and Ferroelectric Properties

**Figure 3:**
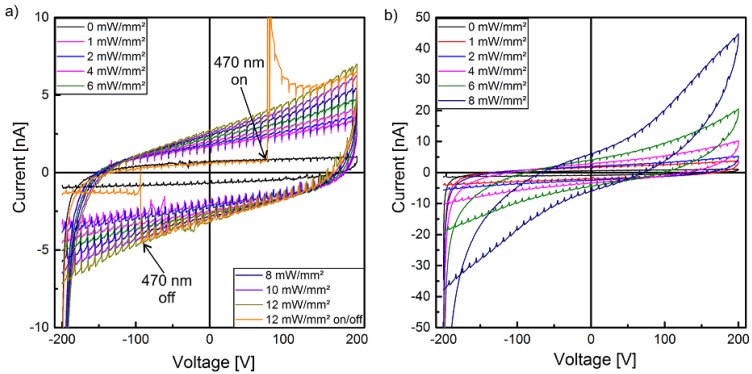
I-V curves of (**a**) sample 1 and (**b**) sample 2 for various optical excitation intensities.

Figure 3 shows the I-V characteristics of both samples. The graphs indicate very similar electrical properties up to an excitation intensity of 2 mW/mm^2^.

With increasing optical power, the I-V characteristic becomes increasingly nonlinear. This effect is more pronounced in sample 2, where I increases up to almost $5\times 10^{-8}$ A at 8 mW/mm^2^ (Figure 3b). The increase of the current is smaller than the increase of the optical excitation intensity but becomes stronger above 2 mW/mm^2^, especially for sample 2. Figure 3a also shows that the conductivity of the samples responds immediately on the optical excitation (470 nm on) and its switch-off (470 nm off).

**Figure 4:**
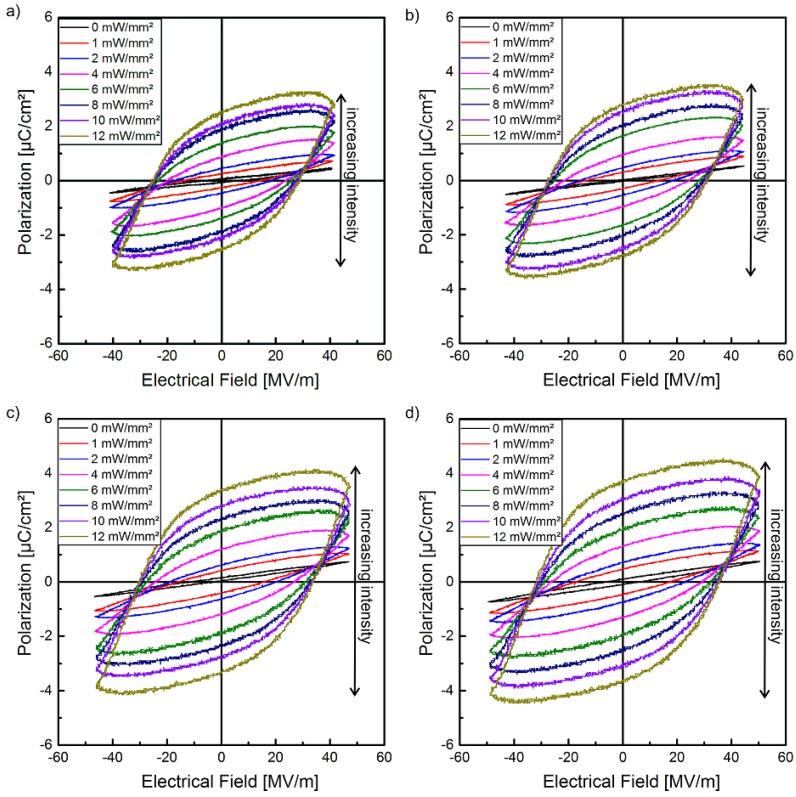
Hysteresis loops of the polarization of sample 1 in dependence of the optical excitation intensities at different peak-to-peak voltages: (**a**) 2.8 kV, (**b**) 3.0 kV, (**c**) 3.2 kV, and (**d**) 3.4 kV.

The hysteresis loops of the polarization P of sample 1 in dependence of the electrical field E with different optical excitation intensities at the wavelength of 470 nm are shown in Figure 4. The hysteresis curves are displayed as measured, i.e., leakage currents were not subtracted. In combination with the supplementary measurements of the pyroelectric coefficient and the polarization profile (LIMM), the polarization of the composite can be clearly demonstrated. In contrast to the pristine polymer with a high crystallinity, for all electric field amplitudes used here, a polarization hysteresis of the composite cannot be observed without an optical excitation. This behavior can be explained by several effects. On the one side, the (Cd:Zn)S-particles decrease the volume fraction of the crystalline *β*-phase and their high resistivity causes a reduction of the electric field in the polymer matrix. On the other side, the stabilizing effect with regard to polarization of the crystalline *β*-phase and therefore of the entire sample due to the compensation polarization of the amorphous phase of P(VDF-TrFE) based on its polarizability, as well as the contributions from dipoles nearby, decrease with increasing particle concentration [49]. Therefore, it is possible to realize homogenous samples that have no ferroelectric hysteresis without optical excitation in a specific range of the electrical field. Moreover, Figure 4 shows that optical excitation not only influences polarization in the sense of an on/off behavior. It can also be utilized to adjust the degree of polarization via the strength of the optical excitation. As illustrated, the polarization hysteresis increases with increasing optical excitation for all utilized peak-to-peak voltages from 2.8 to 3.4 kV (Figure 4).

**Figure 5:**
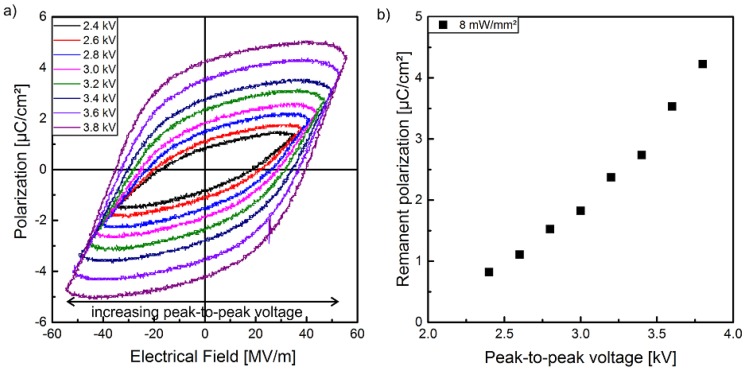
(**a**) Hysteresis loops of the polarization of sample 1 in dependence on the utilized peak-to-peak voltage at an optical excitation intensity of 8 mW/mm^2^ and (**b**) the remanent polarization measured with a combined Sawyer–Tower and pyroelectric coefficient detection setup.

Between each polarization step in Figure 4, the sample had to be demounted from the experimental polarization setup followed by installing into the pyroelectric setup (Figure 1a) and vice versa. Therefore, the pure influence of the electrical field strength was determined for sample 1 at an optical excitation intensity of 8 mW/mm^2^ separately (Figure 5a). In this way it was ensured, that no change of the optical excitation conditions or the measurement setup in general could take place. Furthermore, the additional implementation of the polarization setup in the setup for the determination of the pyroelectric coefficient p allows to measure its temporal behavior directly after polarization. However, this combined experimental setup was limited to a fixed optical excitation intensity, since a necessary permanent control of the optical powersetting could not be achieved experimentally. Similar to the well-known behavior of ferroelectric polymers with no optical dependency like pristine P(VDF-TrFE), the hysteresis loop increases with increasing voltage (here at a peak-to-peak voltage from 2.4 to 3.8 kV) at an optical excitation intensity of 8 mW/mm^2^ (Figure 5a). In contrast to the normal linear or sublinear increase of the hysteresis loops and the remanent polarization with increasing peak-to-peak voltage up to a saturation, a non-linear dependency has been determined with an overlinearly increasing remanent polarization (Figure 5b). This relation can be explained by the influence of the leakage current on the polarization hysteresis, which were detected by the Sawyer–Tower circuit. These leakage currents can result in a more extended hysteresis loop with an overlinearly increasing remanent polarization. This explanatory approach is supported by the increasingly non-linear behavior of the I–V characteristic of sample 1 (Figure 3a) with increasing voltage. Consequently, it can be assumed that the superlinear increase of the photocurrent with higher voltage is also valid for the utilized polarization peak-to-peak voltage range of 2.4 to 3.8 kV, resulting in a non-linear part of the leakage current during the polarization process. This allows the explanation of the non-linear relation between the measured remanent polarization and the peak-to-peak voltage (Figure 5b), but has to be confirmed by the measured pyroelectric coefficient in Section 3.2, which should be a linear or sublinear function of the utilized peak-to-peak voltage.

### 3.2. Pyroelectric Properties

**Figure 6:**
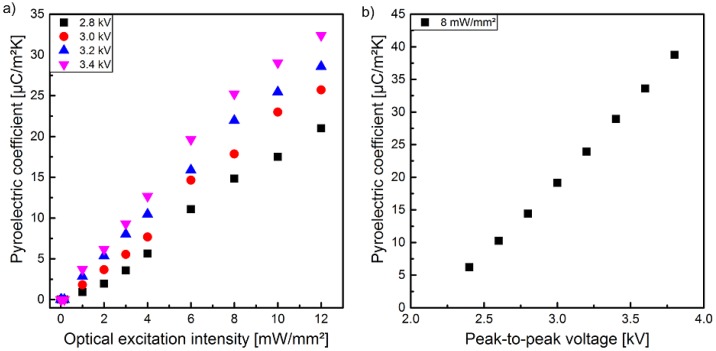
Pyroelectric coefficient of sample 1 in dependency of (**a**) optical excitation intensities at different peak-to-peak voltages and of (**b**) peak-to-peak voltages at an optical excitation intensity of 8 mW/mm^2^ measured with a combined Sawyer-Tower and pyroelectric coefficient detection setup.

**Figure 7:**
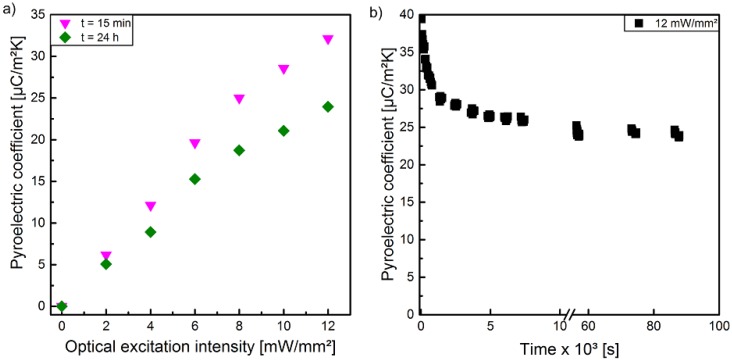
(**a**) Pyroelectric coefficient of sample 1 polarized with a peak-to-peak voltage of 3.4 kV in dependence of the optical excitation intensity during poling measured 15 min and 24 h after poling. (**b**) Decay of the pyroelectric coefficient of sample 1 polarized with a peak-to-peak voltage of 3.4 kV at an optical excitation intensity of 12 mW/mm^2^ over time, measured with a Sawyer–Tower and pyroelectric coefficient detection setup.

In Figure 6a, the pyroelectric coefficient *p* is illustrated as a function of the optical excitation intensity measured for sample 1 polarized at different peak-to-peak voltages. After each polarization step, sample 1 was transferred to the pyroelectric measurement setup with the help of a transfer foil. To ensure a complete thermal adaption of the sample to the temperature of the Peltier element, a certain period of time has been waited for. This time window of approximately 15 min in total (transfer and thermal adaption) ensured that no additional temperature modulation due to temperature adjustment occurred. The pyroelectric coefficient p exhibits a dependency of the optical excitation intensity as well as of the utilized peak-to-peak voltage similar to the polarization measured with the hysteresis loops (Figure 4). Thus, a more or less linear correlation of p to optical excitation intensity as well as to peak-to-peak voltage exists. A comparison of the values of *p* in Figure 6a,b with the same optical excitation and peak-to-peak parameters reveals that the values of *p* in Figure 6b are slightly higher than the values of *p* in Figure 6a. The main difference between these two measurements was the time delay between poling and the measurement of the pyroelectric coefficient *p*. As already mentioned, the peak-to-peak voltage dependent polarization of sample 1 during optical excitation with 8 mW/mm^2^ were performed directly using the pyroelectric measurement setup to prevent demounting and its associated disturbance. This eliminates the need for a transfer between the experimental setups of polarization and pyrolectric coefficient, and thus reduces the time delay by about 10 min. This temporal decay of the pyroelectric coefficient *p* could be confirmed for several optical excitation intensities and a peak-to-peak voltage of 3.4 kV at a time delay of 24 h in contrast to the common delay of 15 min. (Figure 7a). The detailed temporal decay of the pyroelectric coefficient *p* was determined with the combined measurement setup, which implements the polarization setup into the pyroelectric ones. This facilitates a more detailed recording of the decreasing pyroelectric coefficient *p* immediately after the polarization step. Figure 7b shows the exponential decrease and reveals an almost constant behavior after 24 h ($p=25\;\mu C/(m^{2}\cdot K$)).

### 3.3. Thermal Properties and Pyroelectric Depth Profile

**Figure 8:**
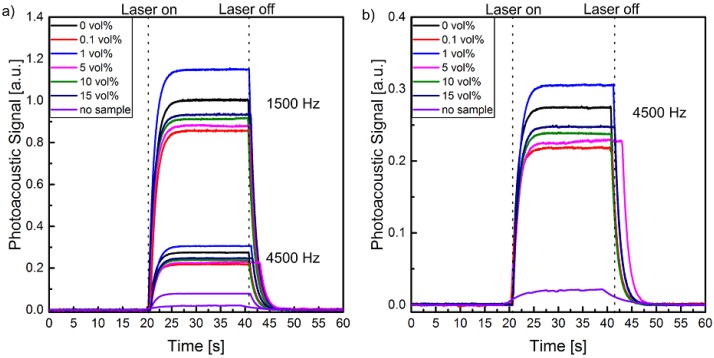
PA signal of P(VDF-TrFE) composite films with different (Cd:Zn)S particle concentrations normalized to the 1500 Hz PA-signal of pristine P(VDF-TrFE) (0 vol %) at (**a**) 1500 Hz, 4500 Hz, and (**b**) 4500 Hz magnified.

Figure 8 shows the relative PA signals of the composite samples with particle concentrations from 0 to 15 vol % as a function of time. The curves are normalized to the absolute PA signal of the pristine polymer sample (0 vol %) at 1500 Hz (Figure 8a).

It becomes evident that there is a maximum difference in the signal amplitudes of the various samples of about 15%. As known, the SA decreases with increasing frequency [44], but also at *f* = 4500 Hz the relationship between the composite samples and pristine polymer remains the same (Figure 8b). This means on one hand that at the utilized frequency of *f* = 1500 Hz the brass cylinder has no further influence on the thermal behavior of the experimental system except for the reduction of the PA cell volume. On the other hand, the samples seem to be homogenous in the considered thickness range (5 µm for 1500 Hz and 2.7 µm for 4500 Hz). This is confirmed by the low SA of the pure brass cylinder without a sample, which is only 10% of the pristine composite sample (Figure 8). No systematic correlation between the PA-signal strength and the particle concentration is observed. The differences in the PA-signal amplitudes between the samples may be caused by sample-specific surface roughnesses in the nm- and µm-range, which lead to variations of the reflectance and therefore also of the absorption of the thin gold layer [50]. Within the experimental precision of the performed PA experiments there is no significant difference in the thermal transport properties of the utilized samples with (Cd:Zn)S particle concentrations between 0 and 15 volume %. This applies in particular with regard to the required accuracy of the thermal properties for the LIMM measurements, due to the relatively broad thermal scanning function [30].

**Figure 9:**
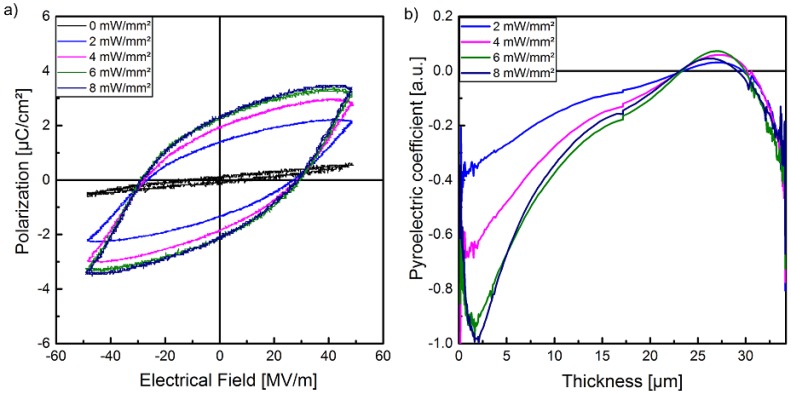
(**a**) Hysteresis loops of sample 2 at a peak-to-peak voltage of 3.4 kV for various optical excitation intensities and (**b**) the corresponding pyroelectric depth profiles resulting from LIMM measurements.

In Figure 9a the ferroelectric hysteresis of sample 2 for a peak-to-peak voltage of 3.4 kV is shown. Similar to sample 1 (Figure 4), at this peak-to-peak voltage no polarization is detected without optical excitation. Hysteresis is observed under illumination and the hysteresis loops become wider with increasing optical excitation intensity. The maximum remanent polarization $P_{r}$ of about $2.4\;\mu C/{\mathrm{cm}}^{2}$ is achieved with an optical excitation intensity of 6 mW/mm^2^. There is no essential change in the shape of the hysteresis curves when the optical excitation intensity is further increased to 8 mW/mm^2^. After each polarization process a waiting time of 24 h was inserted to ensure that the pyroelectric coefficient and its profile have reached steady state (see Section 3.1, Figure 7b) before LIMM measurements were performed.

LIMM measurements were performed from both sides of the sample (in Section 2.3). Based on the results of the photoacoustic measurements, the data of specific heat capacity and thermal conductivity of the pristine polymer have been used for the evaluation of the profiles. A smooth assembly of the two profiles from each side is achieved in a distance of 19 µm from each side, i.e., for an assumed thickness of 38 µm. The discrepancy of 4 µm to the real thickness of the sample is caused by the broad thermal scanning function in the middle of the sample [30]. The pyroelectric depth profiles, normalized to the real thickness of 34 µm, obtained after poling under illumination are shown in Figure 9b. No pyroelectric activity was recorded after the poling step without optical excitation. All depth profiles show a maximum pyroelectric coefficient at the sample side which was exposed to optical excitation during the polarization process (0 µm). The maximum is very close to the sample surface for an optical excitation intensity of 2 mW/mm^2^ and shifts slightly away from the surface for higher intensities and amounts to approximately 2 µm for 6 mW/mm^2^ and 8 mW/mm^2^. From these maxima the pyroelectric activities decreases in the direction towards the bulk of the film. At the non-illuminated sample side the polarization depth profiles look similar for all optical excitation intensities. They show a drop over a distance of about 6 µm and a reversal of the sign over further 5 µm.

It was not possible to measure absolute values of the pyroelectric coefficient as sample 2 was mounted on the special circuit board for LIMM measurements (see chapter 2.1). Nevertheless, on the basis of the LIMM measurements, the relative pyroelectric coefficients of the sample polarized under the different optical excitation intensities can be compared. After poling under optical excitations of 6 and 8 mW/mm^2^, almost identical LIMM signal amplitudes have been detected for a modulation frequency of 10 Hz at which the sample is heated uniformly, regardless of whether the front or rear side was irradiated. This indicates that the pyroelectric coefficient is the same. Furthermore, the pyroelectric depth profiles determined by LIMM-measurements are almost the same for samples polarized under these two optical excitations (Figure 9b).

## 4. Discussion

In the present study, the electrical, ferroelectric and pyroelectric properties of composite films with 10 vol % (Cd:Zn)S particles dispersed in P(VDF-TrFE) have been investigated with respect to the influence of an optical excitation during poling. It was observed that optical excitation causes electrical conductivity which increases with intensity (Figure 3). On the time scale of the experiment the current and thus the conductivity of the composite changes immediately, i.e., without recordable time delay after the optical excitation is switched on or off (Figure 3a).

**Figure 10:**
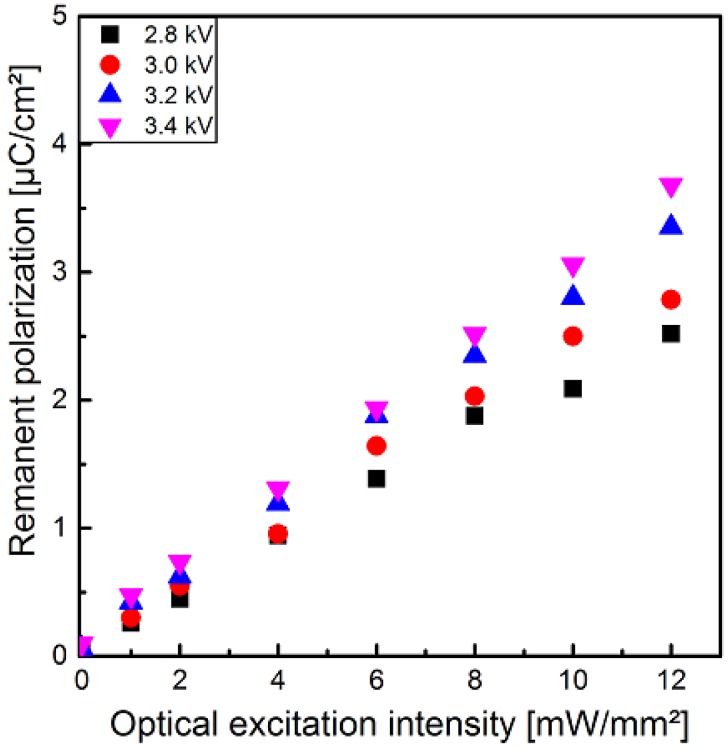
Remanent polarization of sample 1 as a function of optical excitation intensities at different peak-to-peak voltages.

Furthermore, the photoconductivity depends non-linearly on the applied voltage, which particularly becomes evident (in sample 2) for higher excitation intensities exceeding 6 mW/mm^2^. The discrepancy in the I-V characteristics might be the result of a slightly inhomogeneous dispersion of the particles, which causes different conductivities. This was also observed in our previous work [28], where only two instead of four layers of composite films were used to prepare the samples (chapter 2.1). Nevertheless, the use of more than two layers allows the reduction of the conductivity with the same semiconductor particle fraction of 10%. The non-linear I–V characteristic under photo excitation, especially at higher voltages, may be related to carrier hopping and/or tunneling effects occurring at semiconductor– or metal–polymer interfaces [51]. However, the samples in the present study with a conductivity reduced by at least one order of magnitude when compared to our previous work [28], show no polarization hysteresis (Figure 4) without optical excitation at the utilized peak-to-peak voltages. This dependence of the P-E hysteresis loops on the optical excitation intensity also results in an increase of the remanent polarization with increasing optical excitation (Figure 10). As already known, the remanent polarization also depends on the maximum electrical field and increases with increasing peak-to-peak voltage (Figure 10).

**Figure 11:**
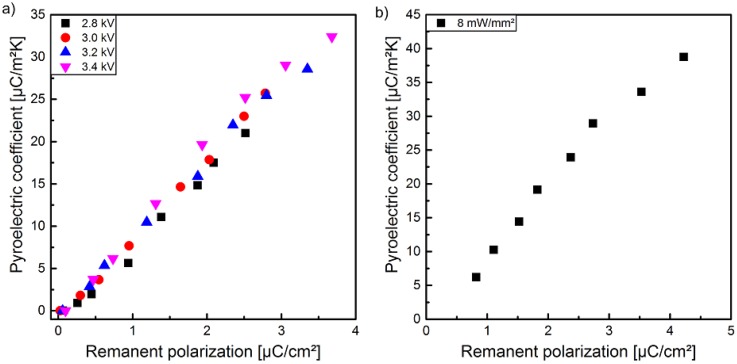
Pyroelectric coefficient of sample 1 (**a**) as a function of the remanent polarization at different peak-to-peak voltages and (**b**) as a function of the remanent polarization at an optical excitation intensity of 8 mW/mm^2^ during the polarization measured with a combined Sawyer–Tower and pyroelectric coefficient detection setup.

The pyroelectric coefficient recorded 15 min after the polarization process increases almost linear with the optical excitation intensity (Figure 6a) as well as the remanent polarization from the hysteresis curves (Figure 11a), confirming the observation in [28], i.e., the observed widening of the hysteresis curves with increasing optical excitation is not caused by a leakage current [24]. For larger electrical fields (3.4 kV) the pyroelectric coefficient shows an additional slight increase when compared to smaller electrical fields (2.8 kV) at the same remanent polarization. Furthermore, a deviation from the almost linear relationship between the pyroelectric coefficient and the remanent polarization begins at peak-to-peak voltages exceeding 3.4 kV (Figure 11b). This might be explained by the increasing influence of the photo excited leakage current on the polarization hysteresis at higher peak-to-peak voltages measured with the Sawyer-Tower circuit [52].

The observed (nonlinear) dependence of the conductivity on the optical excitation intensity and the external voltage amplitude may be caused by space charge regions inside the material, similar to space charge limited currents in semiconductors and electrets [53]. Here, charge carriers are injected into the material during the poling process [54]. Thus, the composite is in a thermodynamically metastable state immediately after polarization, which is not stable with time (Figure 7), because the additional charge carriers rearrange or diffuse out of the material under short circuit conditions [54,55,56]. This results in a decrease of the measured pyroelectric coefficient, since the space charge regions initially lead to an increased polarization. This additional pyroelectric term disappears after the diffusion of the additional charge carriers out of the sample or after their recombination (Figure 7). Subsequently, the pyroelectric coefficient remains almost constant (Figure 7). For this reason, the LIMM measurements were performed 24 h after the polarization, in order to characterize the steady-state condition and to avoid a temporal change of the pyroelectric profiles during the LIMM measurement (see chapter 3.3).

**Figure 12:**
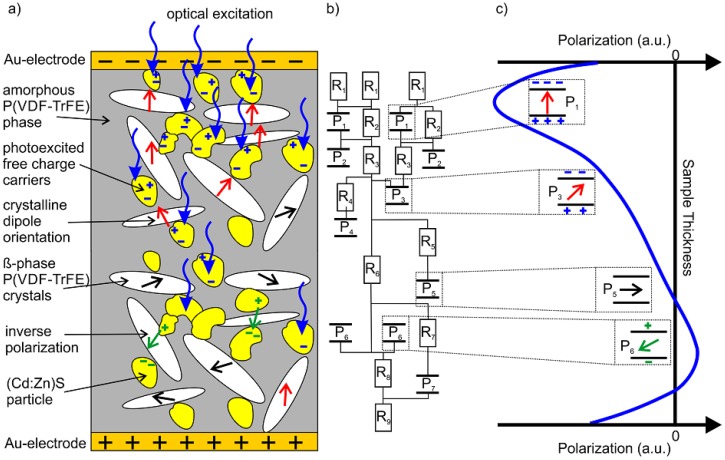
Schematic illustrations of (**a**) the extended “three phase model” that bases on [28], (**b**) the resulting replacement circuit diagram with it’s optical excitation intensity dependency for both, the local resistivity of the sub-composite consisting of amorphous P(VDF-TrFE) and (Cd:Zn)S semiconductor particles (R_1_ < R_2_ <… < R_9_) as well as the local polarization of the crystalline *β*-phase (P_1_ < P_2_ < P_3_ < P_4_ < P_5_ < P_6_ > P_7_), and (**c**) the resulting polarization profile over the sample thickness under large optical excitation conditions.

The pyroelectric depth profiles resulting from poling under optical excitation (Figure 9b) in combination with the results obtained for the I-V characteristic, the P-E hysteresis loops, the remanent polarization, and the pyroelectric coefficient are in accordance with predictions from the “three phase model” presented in [28] (Figure 12a). Furthermore, the experimental results presented here allow an improvement of the model. This “three phase model” is based on two local effects inside the composite samples. Firstly, this is the enhanced electric field in the P(VDF-TrFE) crystal *β*-phase. Secondly, the (Cd:Zn)S photoconductor particles dispersed in the amorphous P(VDF-TrFE) allow an efficient transport of compensating charges to the interfaces with the P(VDF-TrFE) crystallites. This charge transport is directly controlled by the incident light.

The pyroelectric profiles with maxima of the pyroelectric coefficient close to the illuminated surface and its decrease into the bulk (Figure 9b) illustrate the role of illumination during poling. The light intensity decreases from the illuminated surface into the bulk as described by Lambert–Beer’s law and so does the photoconduction in the (Cd:Zn)S particles. Photoconduction enhances the local electric field in the P(VDF-TrFE) crystallites [24,49] and facilitates poling. Furthermore, the charges generated by the photoconducting particles stabilize the polarization of the P(VDF-TrFE) crystallites. As a result the light intensity profile during poling is indicated in the resulting pyroelectric profiles. When the optical excitation intensity exceeds 4 mW/mm^2^ photoconduction increases charge injection from the electrodes into the sample and the electric field in the P(VDF-TrFE) crystallites is no longer maximum at the illuminated surface. As a consequence the maximum in the pyroelectric profile is no longer located directly at the electrode surface but shifts inside the material (2 µm at an excitation intensity of 6 mW/mm^2^). This is probably a result of strong local excitation of additional charge carriers at the sample surface. As a result the surface near region shows a strong increase in the conductivity, resulting in a shift of the maximum effective electric field into the sample. A further increase of the optical excitation intensity up to 8 mW/mm^2^ results on the one hand in additional charge carriers. On the other hand, the global resistance of the sample decreases as seen in the drastically increasing leakage current (Figure 3b) without any further increase in the local electric field. Consequently, the polarization profiles resulting from excitation intensities of 6 mW/mm^2^ and 8 mW/mm^2^ are almost identical.

The effect of carrier injection resulting in a locally increased electrical field inside a ferroelectric sample and the stabilization of this polarization due to space charge regions is well known from “thermally poled” Polyvinylidene fluoride (PVDF) [57]. However, at “thermal polarization” the injection of carriers is realized by the poling conditions at high temperatures (>90 °C) and long periods of time (>1 s). This means the electrical and ferroelectric properties vary homogeneously over the entire thickness of the sample due to its homogeneous heating. As a consequence, the polarization profile of “thermally polarized PVDF” is usually symmetric, starting with a maximum in the center of the sample. This is obviously not the case with sample 2 used here (Figure 9b), as the local resistivity is inhomogeneous over the sample depth due to the inhomogeneous optical excitation (Lambert–Beer’s law) (Figure 12b). Based on the “three phase model”, this leads to an inhomogeneous and non-symmetrical pyroelectric and thus also polarization depth profile (Figure 12c). The decreasing local conductivity with increasing distance from the illuminated surface (Figure 12b, R_1_ < R_9_) of sample 2 might also contribute to the change in sign of the local pyroelectric coefficient observed in a certain range of the sample thickness (from about 22 to 28 µm) (Figure 12b,c, P_6_). As a consequence of the non-linear I-V behavior and the decreasing local conductivity with increasing sample depth (Lambert–Beer’s law) additional injected charge carriers remain in a certain depth of the sample (in the range of 25 µm) after switching off the polarization voltage. Starting from this space charge zone, there is an inverse polarization direction in comparison to the originally applied external electric field [58]. These space charge accumulation is certainly spatially limited and its local field strength is much smaller than the initial external electric field, so that the polarization direction is reverted only over a small thickness range [58].

## 5. Conclusions

The electrical and ferroelectric properties of composite films consisting of 10 vol % (Cd:Zn)S particles in a P(VDF-TrFE) matrix have been investigated regarding the influence of different optical excitation intensities at a central wavelength of 470 nm during poling. Pyroelectric measurements confirmed, that the poling of homogeneous composites is not possible without optical excitation at the utilized (comparably) small peak-to-peak voltages. In addition, it was shown that the degree of polarization can be adjusted by the intensity of the optical excitation in combination with the applied electric field. Polarization depth profiles obtained from LIMM measurements allow a qualitatively improved extension of the previously described “three phase model” [28], with the approach of spatially dependent conductivity and polarization. This is not only important with regard to a refined explanation model that results in a potential replacement circuit diagram. It also demonstrates the use of optical excitation by a laser as an additional tool to realize and control particular polarization distributions in films with a semitransparent electrode that extends over the entire sample surface. Future studies based on the extended “three phase model” may offer the possibility of a targeted modulating of polarization profiles by optical excitation.
